# Two evolutionarily conserved sequence elements for Peg3/Usp29 transcription

**DOI:** 10.1186/1471-2199-9-108

**Published:** 2008-12-10

**Authors:** Jeong Do Kim, Sungryul Yu, Jung Ha Choo, Joomyeong Kim

**Affiliations:** 1 Department of Biological Sciences, Louisiana State University, Baton Rouge, LA, 70803, USA

## Abstract

**Background:**

Two evolutionarily Conserved Sequence Elements, CSE1 and CSE2 (YY1 binding sites), are found within the 3.8-kb CpG island surrounding the bidirectional promoter of two imprinted genes, *Peg3 *(Paternally expressed gene 3) and *Usp29 *(Ubiquitin-specific protease 29). This CpG island is a likely ICR (Imprinting Control Region) that controls transcription of the 500-kb genomic region of the *Peg3 *imprinted domain.

**Results:**

The current study investigated the functional roles of CSE1 and CSE2 in the transcriptional control of the two genes, *Peg3 *and *Usp29*, using cell line-based promoter assays. The mutation of 6 YY1 binding sites (CSE2) reduced the transcriptional activity of the bidirectional promoter in the *Peg3 *direction in an orientation-dependent manner, suggesting an activator role for CSE2 (YY1 binding sites). However, the activity in the *Usp29 *direction was not detectable regardless of the presence/absence of YY1 binding sites. In contrast, mutation of CSE1 increased the transcriptional activity of the promoter in both the *Peg3 *and *Usp29 *directions, suggesting a potential repressor role for CSE1. The observed repression by CSE1 was also orientation-dependent. Serial mutational analyses further narrowed down two separate 6-bp-long regions within the 42-bp-long CSE1 which are individually responsible for the repression of *Peg3 *and *Usp29*.

**Conclusion:**

CSE2 (YY1 binding sites) functions as an activator for *Peg3 *transcription, while CSE1 acts as a repressor for the transcription of both *Peg3 *and *Usp29*.

## Background

*Peg3 *was the first imprinted gene to be identified in proximal mouse chromosome 7, and encodes a C2H2-type zinc finger protein, which is predicted to be a DNA-binding transcription factor [1]. The expression of *Peg3 *is ubiquitous, spatially and temporarily, but exhibits very high levels in the neuronal and germ cells of both mouse and human [2,3]. Consistently, *Peg3 *has been shown to be involved in controlling nurturing behaviors and fetal growth rates [4]. Independent studies also indicated that human *PEG3 *might be a downstream gene for the p53-mediated apoptosis pathway [5,6]. *Usp29 *is another imprinted gene that is located right next to *Peg3 *in a head-to-head orientation [3]. The physiological role of *Usp29 *is currently unknown, but it is believed to be involved in the ubiquitin-mediated protein degradation pathway. According to previous studies, *Usp29 *is also expressed highly in mouse brains but much lower levels than *Peg3 *[3]. The overall spatial expression patterns of *Usp29 *are similar to those of *Peg3*. The transcription start sites (TSSs) for *Peg3 *and *Usp29 *are located very close to each other, 150 bp apart, and thus a genomic region surrounding the two TSSs is thought to function as a bidirectional promoter for *Peg3 *and *Usp29*. A broader genomic region, 3.8 kb in length, surrounding the two TSSs is a CpG island displaying allele-specific DNA methylation. The maternal allele of this region is methylated while the paternal allele is unmethylated [7]. Thus, this region is called the Peg3-DMR (Differentially Methylated Region). This allele-specific methylation pattern is setup during gametogenesis and maintained throughout somatic cells [8]. This maternal-specific methylation of the Peg3-DMR is consistent with the paternal-specific expression of the two genes.

**Figure 1:**
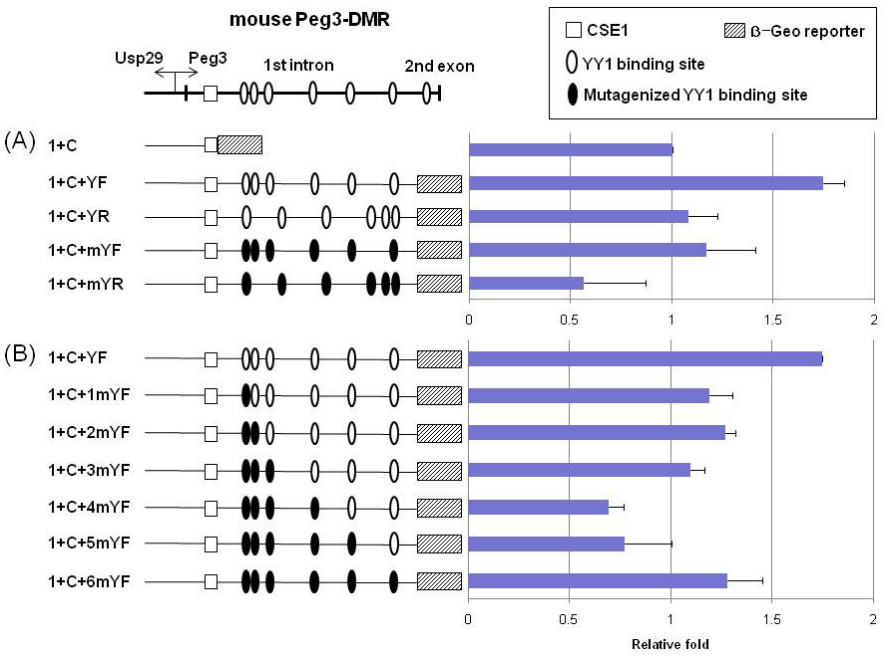
**CSE2 (YY1 binding sites) functions as a transcriptional activator**. The top panel shows the genomic layout of the mouse Peg3-DMR along with the two evolutionarily conserved sequence elements, the CSE1 (open square) and the YY1 binding sites (open oval). The thick vertical lines indicate the positions of the 1^st ^and 2^nd ^exons of *Peg3*. Each of the constructs differs from the others by the orientation and number of YY1 binding sites: the intact versus mutated YY1 binding sites (-Y or -mY), the orientation of the YY1 binding sites (-F or -R), and the numbers of mutated YY1 binding sites (-1 mY to -6 mY) (A&B). Black ovals represent mutated YY1 binding sites. The promoter activity of each construct was analyzed more than three times, and the averaged value was derived along with S.D. (Standard Deviation). The averaged value for each construct was further compared with that of the reference constructs. The two sets of promoter assays were conducted at two different times with two different reference constructs: Construct 1+C (A) and Construct 1+C+YF (B). In order to compare the results from these two different series, the averaged value for the reference construct (1+C+YF) of the second set was adjusted to 1.7 since the same construct derived the 1.7 fold relative to the reference construct (1+C) of the first set. These promoter assays were performed using three different cell lines, Neuro2A, NIH3T3, and HeLa. Only the result set from the Neuro2A cell line is shown in the graph since the other two result sets derived from NIH3T3 and HeLa cells showed almost identical patterns [see Additional file 1].

The Peg3-DMR can be divided into two smaller regions: the 1.3-kb promoter region containing two 1^st ^exons and TSSs for *Peg3 *and *Usp29*, and the 2.5-kb 1^st ^intron region of *Peg3 *(Fig. 1). The 2.5-kb 1^st ^intron of *Peg3 *is known to harbor two evolutionarily conserved sequence elements, CSE1 and CSE2 [9]. A single copy of the 42-bp-long CSE1 is located 1-kb downstream of the 1^st ^exon of *Peg3*. The relative position and orientation of CSE1 to Peg3's 1^st ^exon are well conserved among all the mammals tested. The G-rich sequence of CSE1 is also well conserved. However, the exact roles and binding proteins for CSE1 are currently unknown. By contrast, many copies of the 11-bp CSE2 are distributed over the 2.5-kb region without any obvious pattern of location and spacing among different mammals. But the orientation of each copy of CSE2 is the same and is conserved among different species. Similar clusters of transcription-factor binding sites are also observed in other imprinted domains, such as CTCF sites in *H19*/*Igf2 *and *Dlk1*/*Gtl2 *[10-12]. As seen in the CTCF sites of other domains, the genomic region surrounding the CSE2s was also shown to have an enhancer-blocking activity [9,13]. According to ChIP experiments, CSE2 acts as an *in vivo *DNA-binding site for transcription factor YY1. YY1 binding to CSE2 is allele-specific, only to the unmethylated paternal allele of the Peg3-DMR. Subsequent siRNA-based YY1 knockdown experiments further demonstrated the involvement of YY1 in the DNA methylation and transcriptional control of the Peg3-DMR [14,15]. Nevertheless, it is still unclear if and how CSE2 (YY1 binding sites) are directly involved in the transcriptional control and other related roles of the Peg3-DMR.

The two conserved elements, CSE1 and CSE2, are likely to have functional roles in both genomic imprinting and transcription of the two neighbor genes, *Peg3 *and *Usp29*. In the current study, we sought to characterize these two elements in terms of their possible roles in transcriptional control for *Peg3 *and *Usp29 *using *in vitro *reporter assay systems. We performed several series of mutagenesis experiments and analyzed the effects of these mutations on the transcriptional activity of the Peg3-DMR. According to the results, CSE2 (YY1 binding sites) functions as an orientation-dependent transcriptional activator for the *Peg3 *transcription, whereas CSE1 acts as an orientation-dependent repressor for the transcription of both *Peg3 *and *Usp29*.

## Results

### CSE2 (YY1 binding sites) as a transcriptional activator

To analyze the functional roles of CSE2 (YY1 binding sites) in the transcriptional activity of the Peg3-DMR, we have generated a series of 10 different constructs by modifying and subcloning the 3.8-kb endogenous region of the mouse Peg3-DMR containing 6 YY1 binding sites into a promoterless reporter system, IRES-β-Geo (Fig. 1). Each of these constructs differs from the others by the orientation and number of YY1 binding sites. The orientation of the YY1 binding sites was reversed by subcloning the 2.5-kb YY1 binding region in the opposite orientation relative to the endogenous genomic region of the Peg3-DMR (Constructs named as -YR). To mutate the 6 YY1 binding sites of mouse Peg3-DMR, three bases located within the core motif of each YY1 binding site were changed from GGCGCCATCTT to GGCATTATCTT (Constructs named as -mY). This mutagenesis experiment was also performed serially on each of the YY1 binding sites starting from the one located on the 5'-side to the one on the 3'-side, resulting in a series of the fragments containing the different numbers of YY1 binding sites (Constructs named as 1 mYF to 6 mYF in Fig. 1B). These constructs were individually transfected into three different cell lines, HeLa, NIH3T3 and Neuro2A, along with the internal control luciferase vector (pGL3 control) in order to normalize the β-gal activity.

The first series of experiments was conducted as shown in Fig. 1A. In this series, the normalized transcriptional activity of each construct was compared to the activity of the construct 1+C, which contains the 1.3-kb genomic region spanning from the promoter to the CSE1 region of *Peg3*. As compared to the promoter activity of Construct 1+C, the inclusion of the 2.5-kb YY1 binding region in the forward direction resulted in a 1.7-fold increase (Construct 1+C+YF), but not when it was included in the reverse direction (Construct 1+C+YR). This suggests that the observed boosting effect of the 2.5-kb YY1 binding region is orientation-dependent. The constructs containing mutated YY1 binding sites showed a dramatic decrease in the promoter activity of *Peg3 *in both directions (Construct 1+C+mYF or 1+C+mYR). There are only 18 base differences (3 base changes per YY1 binding site × 6 YY1 binding sites) between Construct 1+C+YF and Construct 1+C+mYF. Yet, these targeted mutations on 6 YY1 binding sites completely abrogated the original increase in activity that was caused by the inclusion of the 2.5-kb YY1 binding region. Also, the direction of the mutated YY1 binding sites did not have any major effect on activity (Constructs 1+C+mYF and 1+C+mYR) although the opposite direction somewhat reduced the activity. However, this reduction was not observed consistently in other cell lines [see Additional file 1]. Therefore, the above results suggest that the increased transcriptional activity caused by the 2.5-kb YY1 binding region is mainly derived from these 6 YY1 binding sites.

The second series of experiments was performed to test the effects of different numbers of YY1 binding sites on the transcriptional activity of the Peg3-DMR (Fig. 1B). In this series, the normalized transcriptional activities of all the constructs were compared to the activity of the construct 1+C+YF, which contains 6 intact YY1 binding sites. The value for this control construct was adjusted to 1.7, which is the estimated value from the first series, in order to compare the different values derived from the two different series (Fig. 1A and 1B). As shown in Fig. 1B, the transcriptional activities of all constructs decreased as compared to that of the construct 1+C+YF. The mutations on the first three YY1 binding sites resulted in about 30% reduction in the activity (1+C+1 mYF, +2 mYF, +3 mYF), and the mutations on two additional YY1 sites further decreased the activity (1+4 mYF and +5 mYF). The observed decrease in the transcriptional activity of the Peg3-DMR is somewhat gradual but not proportional to the reduction in the number of YY1 binding sites. In particular, the final mutation construct (1+C+6 mYF with no YY1 sites) reversed this overall decreasing pattern with the activity about 80% of the control vector (1+C+YF). This suggests that each of the YY1 binding sites might have different levels of contribution and/or even different roles. The results presented in Fig. 1B are derived from IRES-β-Geo, but another set of results derived from the more sensitive IRES-Luciferase system also derived an almost identical pattern (data not shown). In sum, the above results demonstrated the YY1 binding sites as a transcriptional activator for the Peg3-DMR, and further suggest that each of the YY1 binding sites may have slightly different levels of contribution and/or role for the overall transcriptional activity of the Peg3-DMR.

The above results were consistently observed from three different cell lines that have been used for these promoter assays. We are providing the results from HeLa and NIH3T3 [see Additional file 1]. The above sets of experiments were also repeated to test the potential roles of CSE2 in the *Usp29 *direction. However, we were not able to detect any transcriptional activity of the bidirectional promoter in the *Usp29 *direction (data not shown).

### CSE2 (YY1 binding sites) not as an enhancer blocker

**Figure 2:**
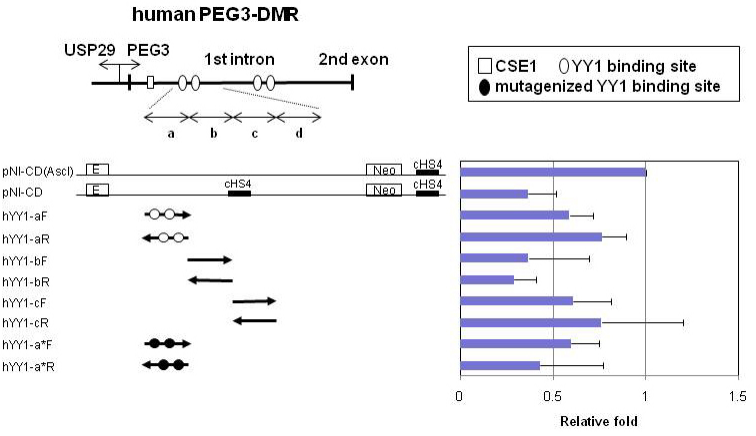
**CSE2 (YY1 binding sites) does not function as an enhancer blocker**. In order to determine the minimal regions for the previously reported insulator activity in human PEG3-DMR [9], the hYY1-1 fragment was further divided into 4 smaller fragments with 200-bp size (hYY1-a through -d), and these fragments were subcloned into the pNI-CD vector to test insulator activity. These inserts were localized between the erythroid-specific enhancer (E) and Neomycin resistance gene (Neo) that is driven by the γ-globulin promoter. The values on the X axis of the graph indicate the fold difference of the survived colony number in the G418 selection relative to the value of the negative control lacking any insulator, pNI-CD(AscI). The last fragment, hYY1-d, did not show any insulator activity, and thus was omitted in this figure. The two YY1 binding sites (oval) within the first fragment, hYY1-a, were mutated and tested again, which is indicated by hYY1-a*. The mutated YY1 binding sites are indicated by black ovals.

Since the genomic region surrounding YY1 binding sites was initially shown to have an insulator (enhancer-blocking) activity, we performed a series of follow-up studies to test if YY1 binding sites are also responsible for this insulator activity [9]. Although the YY1 sites are proven to be an enhancer for *Peg3*, an immediate neighbor gene, it is also possible that YY1 sites may function as an insulator for other distantly located imprinted genes, such as *Zim1 *and *Zim2*, which has been often seen in other imprinted domains. For this series of analysis, we used an original 800-bp DNA fragment derived from human PEG3-DMR, which was used for our initial study [9]. This initial 800-bp fragment of human PEG3-DMR, hYY1-1, was further divided into 4 smaller fragments with approximately 200-bp size in length, hYY1-a through -d (Fig. 2). Two potential YY1 binding sites (GGCGCCATCTT) are located within the first fragment, hYY1-a. The four small fragments were individually analyzed in both orientations for the insulator assay. Among the four small fragments, the second fragment, hYY1-b, showed the most significant and consistent insulator activity, while hYY1-a and hYY1-c also showed some insulator activity but much less than that of hYY1-b. The fourth fragment, hYY1-d, showed almost no insulator activity (data not shown). To further determine the potential involvement of YY1 in the original insulator activity, we mutated the two YY1 binding sites located within the 200-bp sequence of hYY1-a and tested the mutational impact on the insulator activity. As shown in Fig. 2 (hYY1-1a*F and R), mutations on the two YY1 binding sites did not change the insulator activity in either orientation. Also, it is important to note that the second fragment, hYY1-b, does not contain YY1 binding sites and yet showed the most significant insulator activity. This suggests that YY1 is not the transcription factor responsible for the observed insulator activity, and further that unknown regulatory elements other than YY1 might be involved in the insulator activity of the PEG3-DMR.

### CSE1 as a transcriptional repressor for Peg3-DMR

**Figure 3:**
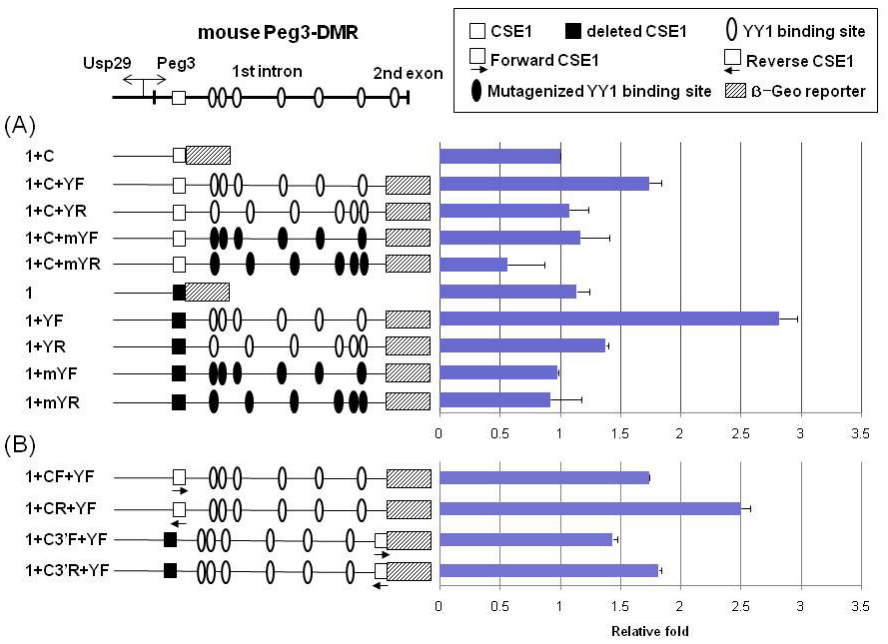
**CSE1 functions as a transcriptional repressor for Peg3-DMR**. The top panel shows the genomic layout of the mouse Peg3-DMR containing the two evolutionarily conserved sequence elements, CSE1 (open square) and YY1 binding sites (open oval). The thick vertical lines indicate the positions of the 1^st ^and 2^nd ^exons of *Peg3*. The transcriptional involvement of CSE1 in mouse Peg3-DMR was analyzed using the IRES-β-Geo promoterless vector system. (A) The first series of assays were conducted with the ten individual constructs that differ by the presence/absence of CSE1 (with or without C in their names), the intact versus mutated YY1 binding sites (-Y or -mY), and the orientation of YY1 binding sites (-F or -R). (B) The second series of assays investigating the position- and orientation-dependency of CSE1 on the *Peg3 *promoter activity were conducted by three additional constructs containing the reverse orientation of CSE1 (-CR & -C3'R) and the 3'-end position of CSE1 (-C3'F & -C3'R). Each construct was analyzed more than three times as shown with the average value with S.D. The averaged value for each construct was compared with that of the reference constructs, and subsequently presented in the graph as fold differences. Two sets of assays were conducted with two different reference constructs: Construct 1+C (A) and Construct 1+CF+YF (B). In order to compare the different values derived from the two different series, the value for the control construct of the second set (1+CF+YF) was adjusted to 1.7 as derived from the first set (1+C+YF). These assays were conducted using three cell lines, Neuro2A, NIH3T3, and HeLa, and they all showed a similar pattern as the above set of assays using the Neuro2A cell line [see Additional file 1].

To test the potential involvement of CSE1 in the promoter activity of the Peg3-DMR, another series of constructs lacking CSE1 were also made and analyzed (Constructs named without C in Fig. 3). The promoter assays with these CSE1-deleted constructs derived a similar overall pattern as the previous CSE1-containing constructs (Fig. 1A): an orientation-dependent transcriptional increase caused by the YY1 binding sites. However, the increase in transcriptional activity resulting from the inclusion of the YY1 binding sites in these CSE1-deleted constructs was much greater than those seen in the CSE1-containing constructs. In particular, Construct 1+YF showed 2.8 fold increase, while Construct 1+C+YF yielded only 1.7 fold increase. This suggests that CSE1 may function as a repressor for the Peg3-DMR. It is also noteworthy that this large increase was observed only in Construct 1+YF, which contains the YY1 binding sites. The constructs lacking the YY1 binding sites, Construct 1 and 1+C, did not show any difference in activity. These results suggest that CSE1 may function as a context-dependent repressor together with an activator function of YY1 binding sites for the promoter activity of the Peg3-DMR.

We further analyzed the position and orientation-dependency of this potential repressor, CSE1, for transcriptional activity of the Peg3-DMR (Fig. 3B). Three modified constructs were generated and subcloned into the IRES-β-Geo promoterless vectors. The orientation of CSE1 of each construct was reversed relative to the direction of *Peg3 *transcription (Constructs named -CR), and the position of CSE1 was also move to the 3'-end of the Peg3-DMR (Construct named -C3'). As shown in Fig. 3B, the construct with the reverse orientation of CSE1 (Construct 1+CR+YF) showed about 1.5 fold more activity relative to the activity of the control (Construct 1+CF+YF). This is similar to the initial boosting effect by the deletion of CSE1 (1+YF in Fig. 3A), confirming the orientation-dependent repressor activity of CSE1. Two constructs with CSE1 in the 3'-end position (constructs 1+C3'F+YF & 1+C3'R+YF) showed slightly less but similar levels of activity than the control (Construct 1+CR+YF), indicating no major impact by changing the relative position of CSE1 to the 3'-end of the Peg3-DMR. Thus, this series of experiments confirms the position-independent repressor role of CSE1. Repositioning CES1 to the 5'-end of the Peg3-DMR was also performed, but this result set was inconclusive mainly due to the fact that the 5'-side region appears to be sensitive to the insertion by any random sequences for subcloning (data not shown). In sum, the above results revealed an orientation-dependent but somewhat position-independent repressor role of CSE1 for the transcriptional activity of the Peg3-DMR.

### Minimal regions of CSE1 for the transcriptional repression of *Peg3 *and *Usp29*

**Figure 4:**
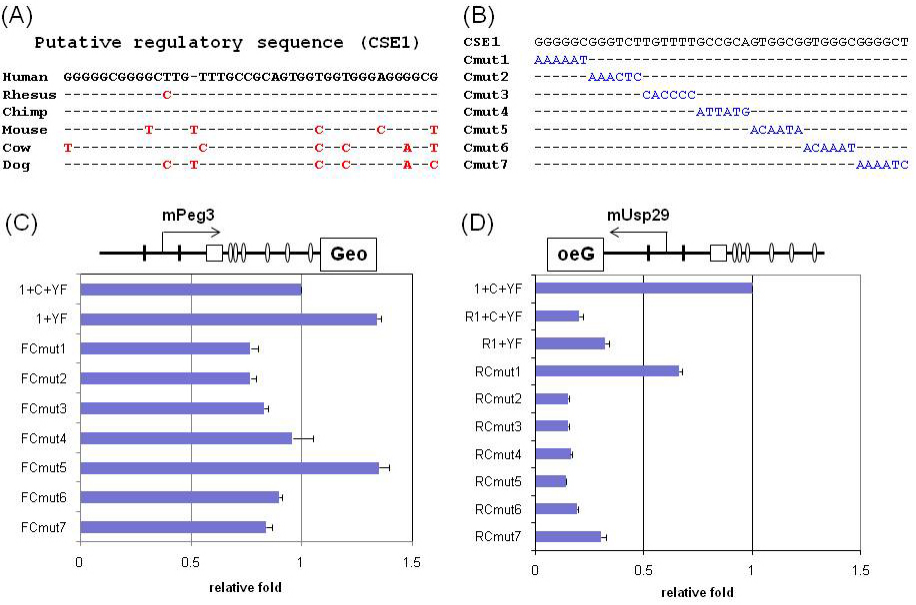
**Minimal regions within the CSE1 responsible for transcriptional repression of *Peg3 *and *Usp29***. (A) Evolutionary conservation of the CSE1 is demonstrated through aligning the sequences derived from six mammals: human, rhesus, chimpanzee, mouse, cow, and dog. The bases in red indicate differences from the human CSE1. The hyphens (-) indicate insertions/deletions. (B) A series of 6-bp-segments were mutated on the mouse CSE1 starting from 5' to the 3'-ends. The intact sequence of the mouse CSE1 is shown on top and the following sequences represent the mutated versions of the CSE1 with the changed bases marked in blue. (C&D) The mutated CSE1 was analyzed for the promoter activity after subcloning into the IRES-β-Geo promoterless vector system. The Peg3-DMR promoter containing a series of mutated CSE1 sequences was analyzed in two different directions: *Peg3 *(C) and *Usp29 *(D) directions. The construct layout on the top region of each graph shows the transcriptional direction of the mouse Peg3-DMR containing CSE1 (open square) and YY1 binding sites (open oval). The thick vertical lines indicate the positions of the 1st exons of *Peg3 *and *Usp29*. Each construct was analyzed more than three times. The averaged value for each construct was first compared with that of the reference construct, in this case Construct 1+C+YF, and the subsequent fold difference was presented with S.D. in the graph. These assays were conducted using two cell lines, Neuro2A and HeLa. The result set shown in the graph was derived from Neuro2A cells but the result set from HeLa cells also showed very similar patterns [see Additional file 1].

The relative orientation and position of CSE1 are well conserved among all the mammals, as shown with a subset of available genome sequences, including human, rhesus, chimpanzee, mouse, cow, and dog (Fig. 4A). This is also consistent with the results observed from the previous experiments, the orientation and YY1 context-dependent nature of this element (Fig. 3). The CSE1 sequences from these species also share high levels of sequence identity, ranging from 86% to 100%, but with a couple of single-base insertions/deletions that are specific to each species. However, the 42-bp-long CSE1 is predicted to be too long for a single DNA-binding protein. Thus, we decided to further define potential minimal regions for the observed repressor activity of CSE1. We have performed serial mutation of 6-bp sections of CSE1 from purine to purine and from pyrimidine to pyrimidine (G ↔ A & T ↔ C) (Fig. 4B). The Peg3-DMR containing each mutated CSE1 was also subcloned into the IRES-β-Geo promoterless vector in both directions (Constructs Cmut1 to Cmut7). The constructs named with F- are positioned in the *Peg3 *direction (Fig. 4C), whereas the constructs with R- are in the *Usp29 *direction (Fig. 4D). These constructs were analyzed in a similar manner as above.

In the *Peg3 *direction, we used two controls: the intact Peg3-DMR (Construct 1+C+YF) and the deletion Peg3-DMR lacking CSE1 (Construct 1+YF). As shown in Fig. 4C, most of the CSE1-mutated constructs showed a slightly lower but similar activity to that of the intact control (Construct 1+C+YF), indicating no major or only marginal effect. However, one construct (FCmut5) showed higher activity than the intact control (Construct 1+C+YF), but similar to the deletion control (Construct 1+YF). This indicates that the 6-bp region mutated within Construct FCmut5 may be solely responsible for the original effect caused by the deletion of the entire region of CSE1 (Fig. 3A). A similar series of experiments was also performed for the *Usp29 *direction (Fig. 4D). Three controls were also used for this series: Construct 1+C+YF for the comparison between the *Peg3 *and *Usp29 *directions, and Construct R1+C+YF (the intact Peg3-DMR) and Construct R1+YF (the deletion Peg3-DMR lacking CSE1). As shown in Fig. 4D, the overall transcriptional activity of the Peg3-DMR is much lower in the *Usp29 *direction than in the *Peg3 *direction (Constructs 1+C+YF vs R1+C+YF). This is true for most of the tested constructs, including the CSE1-mutated constructs as well as the two control constructs (R1+C+YF and R1+YF). Nevertheless, the deletion of CSE1 increased the transcriptional activity based on the observed difference between the two control constructs (R1+C+YF vs R1+YF). This may be an indication that CSE1 still be a potential repressor for the *Usp29 *direction. Interestingly, however, one construct (RCmut1) derived much higher levels of activity than the deletion construct (R1+YF). This is unusual since we have not detected this much transcriptional activity in the *Usp29 *direction using any modified construct derived from the Peg3-DMR. Although this unusual increase requires further investigation, this may represent another critical minimal region for the repression in the *Usp29 *direction. In sum, the above results indicate that CSE1 may function as a repressor for both directions, *Peg3 *and *Usp29*, and also that one minimal region is critical for the *Peg3 *direction.

## Discussion

The results present in the current study demonstrate YY1 binding sites (CSE2) as an orientation-dependent activator for the *Peg3 *transcription, and CSE1 as a repressor for *Peg3 *and *Usp29 *transcription. In addition, two 6-bp minimal regions have been identified within CSE1, one of which most likely plays a critical role for the transcriptional repression in the *Peg3 *direction.

**Figure 5:**
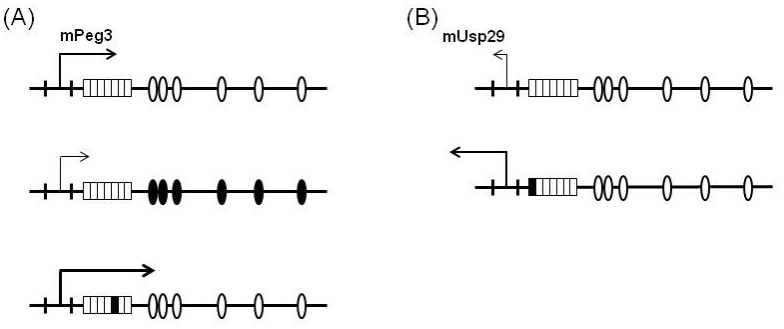
**Potential roles of CSE1 and CSE2 for transcriptional regulation of the *Peg3 *and *Usp29***. Arrows indicate the transcriptional directions of *Peg3 *and *Usp29*, and their size and thickness represent the transcriptional strength of *Peg3 *and *Usp29*. Smaller rectangles indicate seven individual sections within CSE1 that have been individually mutated. The mutated section is marked with a black rectangle. Open and black ovals indicate intact and mutated YY1 binding sites, respectively. In the *Peg3 *direction (A), the YY1 binding sites (CSE2) function as activators, whereas CSE1 acts as a repressor for the *Peg3 *transcription. In the *Usp29 *direction (B), the functional contribution of the YY1 binding sites is unknown, but CSE1 most likely plays a repressor role for *Usp29 *transcription.

The potential roles of two conserved sequence elements in the transcriptional control of *Peg3 *and *Usp29 *could be summarized as depicted in Fig. 5. In the *Peg3 *direction (Fig. 5A), the YY1 binding sites (CSE2) function as an activator while the CSE1 as a repressor for the Peg3-DMR bidirectional promoter. One recent independent study also reported a similar observation but with much less details, highlighting the potential roles of CSE1 and CSE2 for *Peg3 *transcription [16]. The activator role of the CSE2 is orientation-dependent and also somewhat dosage-dependent based on the detection of a gradual decrease in the transcriptional activity of the Peg3-DMR in proportion to the reduction in the numbers of YY1 binding sites (Fig. 1). The repressor role of the CSE1 is also orientation-dependent, but somewhat position-independent (Fig. 3). It is important to note that the repressor role of the CSE1 is only detectible when the YY1 binding sites are present nearby the CSE1. This suggests the presence of a functional, but potential antagonistic, interaction between the two conserved sequence elements, CSE1 and CSE2. In the *Usp29 *direction (Fig. 5B), the functional roles of two elements are not easily discernible mainly due to the very low levels of the activity with *in vitro *reporter assay systems. Thus, the role of the CSE2 in the *Usp29 *direction is currently unknown. On the other hand, the deletion of the CSE1 derived low but consistent levels of increase in the transcriptional activity of the Peg3-DMR in the *Usp29 *direction, suggesting that the CSE1 might also be a repressor for the *Usp29 *direction (Fig. 4B). Furthermore, mutation on one minimal region within the CSE1 showed a dramatic increase in the transcriptional activity, which is higher than the levels of the deletion construct lacking the entire the CSE1 region. This could be an artifact stemming from a serendipitous sequence context generated by mutagenesis experiments, but it could also be a genuine effect reflecting the *in vivo *role of this minimal region. One possible scenario would be that the CSE1 might bind two proteins: one protein with a repressor role bind the first 6-bp minimal region while the other protein with an activator role bind the other minimal region within the CSE1. In that situation, complete deletion would not dramatically increase the transcriptional activity as seen in Construct R1+YF, but mutation of the first 6-bp region would relieve the repression and allow the second protein to activate the transcription in the *Usp29 *direction (Construct RCmut1). Overall, the two *cis*-elements, CSE1 and CSE2, appear to function as a repressor and activator, respectively, for the bidirectional promoter of the Peg3-DMR.

The identification of CSE1 as a repressor is unexpected since the expression patterns and levels of both human and mouse *PEG3 *appear to be ubiquitous and very high compared to those of other genes. Then, what could be biological roles of this unexpected repressor, CSE1, for the *PEG3 *expression? Two scenarios are possible based on all the observations drawn from previous studies. First, CSE1 may be a cell type-specific repressor. Despite high levels of the expression of *PEG3 *in the adult brains of human and mouse, *in situ *hybridization results clearly demonstrated that the spatial expression of *PEG3 *is limited only to a subset of neuronal cells, including cerebellar Purkinje and cerebral pyramidal cells, but not to the other cells, such as cerebellar granule cells [2,3]. CSE1 could be involved in this cell type-specific expression of *PEG3 *in neuronal cells. Second, CSE1 may be a repressor responding to unknown molecular signals for cellular and developmental pathways. According to previous studies, it has been shown that the expression levels of human *PEG3 *are up-regulated by the onset of the p53-mediated apoptosis [5,6]. Also, the expression levels of *PEG3 *are also known to be changed during muscle development as well as by the hypoxia condition in rodent brain cells [17,18]. These observations revealed dynamically changing expression patterns of *PEG3*. CSE1 may be responsible, as a repressor, for these dynamic responses of *PEG3 *to the various conditions and challenges that are faced by normal cells. The above two possibilities remain to be investigated in the near future, but the identification of CSE1 as a repressor provides a new direction for the functional study of *PEG3*.

In contrast to CSE1, the identification of CSE2 (YY1 binding sites) as a transcriptional activator has been expected based on the results derived from previous studies, revealing that similar clusters of YY1 binding sites show an orientation-dependent increase in their transcriptional activity [13]. The current study confirmed, more clearly and precisely, this predicted role of CSE2 through targeted mutations on YY1 binding sites (Fig. 1). However, the activator role of CSE2 is somewhat contradictory to the previous observations derived from siRNA-based YY1 knockdown experiments [14,15]. YY1 is predicted to be a repressor based on the observed up-regulation of *Peg3 *and *Usp29 *against lowering the cellular levels of YY1 protein. These conflicting results warrant further investigation, but may reflect potential differences stemming from the use of two experimental systems. Most of the promoter assays used for this study are episome-based transient experiments, and thus may or may not reflect the *in vivo *role of CSE2 in chromosomal contexts. On the other hand, the effects observed from siRNA-based YY1 knockdown experiments could be easily mired with indirect outcomes. Regardless of as an activator or repressor, CSE2 appears to be very unusual: multiple YY1 binding sites are scattered over the 2.5-kb intron region without any limitations in their spacing and copy numbers. So far, we have not seen any other *cis*-elements for transcription that have such unusual patterns of evolutionary conservation and genomic layout. These unusual features may hint at one possibility that CSE2 is designed for some roles other than direct contribution to transcription, such as setting up and/or maintaining DNA methylation for the Peg3-DMR. Consistently, mutations of the CSE2 appear to have a much milder impact on the transcriptional activity of the Peg3-DMR than mutations of the CSE1, suggesting that CSE1 may be the dominant player in the transcriptional regulation of *Peg3 *and *Usp29*. However, it is important note that CSE1 functions in a context-dependent manner with CSE2 (YY1 binding sites). This suggests the presence of a potential interaction between YY1 and other unknown factors binding to CSE1, and further that this interaction may determine final transcription rates for both *Peg3 *and *Usp29*.

## Conclusion

Taken together, CSE2 (YY1 binding sites) functions as an orientation-dependent transcriptional activator for the *Peg3 *transcription, while CSE1 acts as a repressor for the transcription of both *Peg3 *and *Usp29*.

## Methods

### Plasmids

For the promoter assay of the Peg3-DMR, we have constructed two promoterless vectors containing the IRES-β-Geo (a fusion protein of β-Galactosidase and Neomycin) and luciferase reporters, respectively. The first reporter construct was generated through transferring the 5.1-kb IRES-β-Geo fragment from the pGT1.8Ires-β-geo vector [19] into the BamHI site of pBluescript SK(-). The second reporter, a promoterless IRES-Luciferase, was constructed through transferring the two following fragments to pBluescript SK(+): a HindIII-SalI fragment containing the luciferase gene from pGL4.10 [luc2] (Promega) and a PstI-EcoRV fragment containing the IRES (Internal Ribosomal Entry Site) from pGT1.8Ires-β-geo.

The genomic fragments containing mouse Peg3-DMR [GenBank: AF105262, AF102110–AF105905] were amplified by PCR, and subcloned into the NotI site of the two promoterless reporters, IRES-β-Geo and IRES-Luciferase. To delete CSE1 from the Peg3-DMR, both flanking regions of CSE1 were individually amplified by PCR (Maxime PCR premix kit, Intron Biotech), ligated, and finally subcloned into the reporter vectors. Also, the relative position and orientation of CSE1 were changed through including the CSE1 sequence as part of one of two oligonucleotide primers for PCR. To mutate six YY1 binding sites of mouse Peg3-DMR, three bases located within the core motif of each YY1 binding site were serially changed from GGCGCCATCTT to GGCATTATCTT. The serial mutations of six base pairs segments of CSE1 were performed in the manner of purine to purine and pyrimidine to pyrimidine (G ↔ A & T ↔ C). These serial mutations were performed using the QuikChange II Site-Directed Mutagenesis Kit (Stratagene).

### Promoter assay

For the promoter assays, NIH3T3 and HeLa cell lines were grown in the DMEM medium (GibcoBRL) and the Neuro2A cell line was maintained in the MEM medium (GibcoBRL). Media were supplemented with 10% fetal bovine serum and 1% antibiotic-antimycotic (GibcoBRL). All cell lines were grown at 37°C in a humidified incubator containing 5% CO2. About 2 × 10^5 ^cells were first plated in each well of six-well plates, and then transfected with reporter constructs on the following day using the GeneJuice transfection reagent (Novagen). The detailed information for the reporter assays is as follow. Each well was transfected with the serum-free medium containing 3 μl of GeneJuice and 1 μg of DNA (0.9 μg reporter vector + 0.1 μg pGL3 Control or pRLSV40 vectors [Promega]). Two days after transfection, the cells were harvested, washed with PBS, and treated with 100 μl of the lysis buffer (0.25 M Tris-HCl at pH 7.8 + 0.1% NP40) for 30 mins at 4°C. The cellular debris derived from the lysis step was removed by centrifugation for 10 min. For the β-galactosidase assay, 30 μl of cell lysates were mixed with the same volume of the 2× β-galactosidase assay buffer (Promega) in a 96-well flat-bottom clear plate (Corning). After the incubation of the plate at 37°C, the assay was terminated with 90 μl of 1 M sodium carbonate. Absorbance was measured at 405 nm using the Wallac 1420 multilabel counter VICTOR3 (PerkinElmer). To monitor transfection efficiency, the β-galactosidase activity was normalized with the luciferase activity. For the luciferase assay, 20 μl of cell lysates were combined with 100 μl of the Luciferase assay reagent (Promega) in a 96-well flat-bottom white plate (Corning). The Dual-Glo Luciferase Assay System (Promega) was used for the assay of the IRES-Luciferase vector system. To monitor transfection efficiency, the firefly luciferase activity was normalized to the *Renilla *luciferase activity. Luminescence was measured using the Wallac 1420 multilabel counter VICTOR3 (PerkinElmer).

### Insulator assay

DNA fragments of interest were cloned into the AscI site of pNI-CD (generous gift from Drs. Gary Felsenfeld and Adam West). Each fragment was cloned in both orientations. The locations of the tested DNA fragments within human BAC CIT-470F8 [GenBank: AC006115] are hYY1-a (161993/161803), hYY1-b (161802/161614), hYY1-c (161613/161400), and hYY1-d (161439/161189). Constructs were transfected into K562 cells by electroporation at 200 V, 1000 mF (double pulse) using a Bio-Rad Gene Pulser II. After a 10 min recovery on ice, cells were plated into RPMI supplemented with 10% FBS, 200 mM L-glut and Pen/Strep. Twenty-four hours post transfection, cells were washed and resuspended in Improved MEM zinc option (GibcoBRL). Cells were plated into 0.3% soft agar with 1050 μg/ml Geneticine (GibcoBRL) and incubated at 37°C for 18–21 days.

## Authors' contributions

JDK designed and constructed all the vectors for the study, and also performed the experiments and wrote the paper. SY performed part of the promoter assays and JHC conducted the mutagenesis experiment of YY1 binding sites. JK provided the original concept of the study, supervised the study, and contributed to writing the paper. All authors read and approved the final manuscript.

## Supplementary Material

Additional file 1 **Transcriptional activity assay**. These results derived from other cell lines, HeLa and NIH3T3, using the same construct set presented in Figs. 1A and 3A(A), Fig. 1B(B), Fig. 3B(C), and Fig. 4C(D). Click here for file
