# Supplementary figures and images for: Two evolutionarily conserved sequence elements for Peg3/Usp29 transcription
Source: BMC Mol Biol. 2008 Dec 10;9:108. doi: 10.1186/1471-2199-9-108 (PMC2615030; doi:10.1186/1471-2199-9-108)

## Slide 1
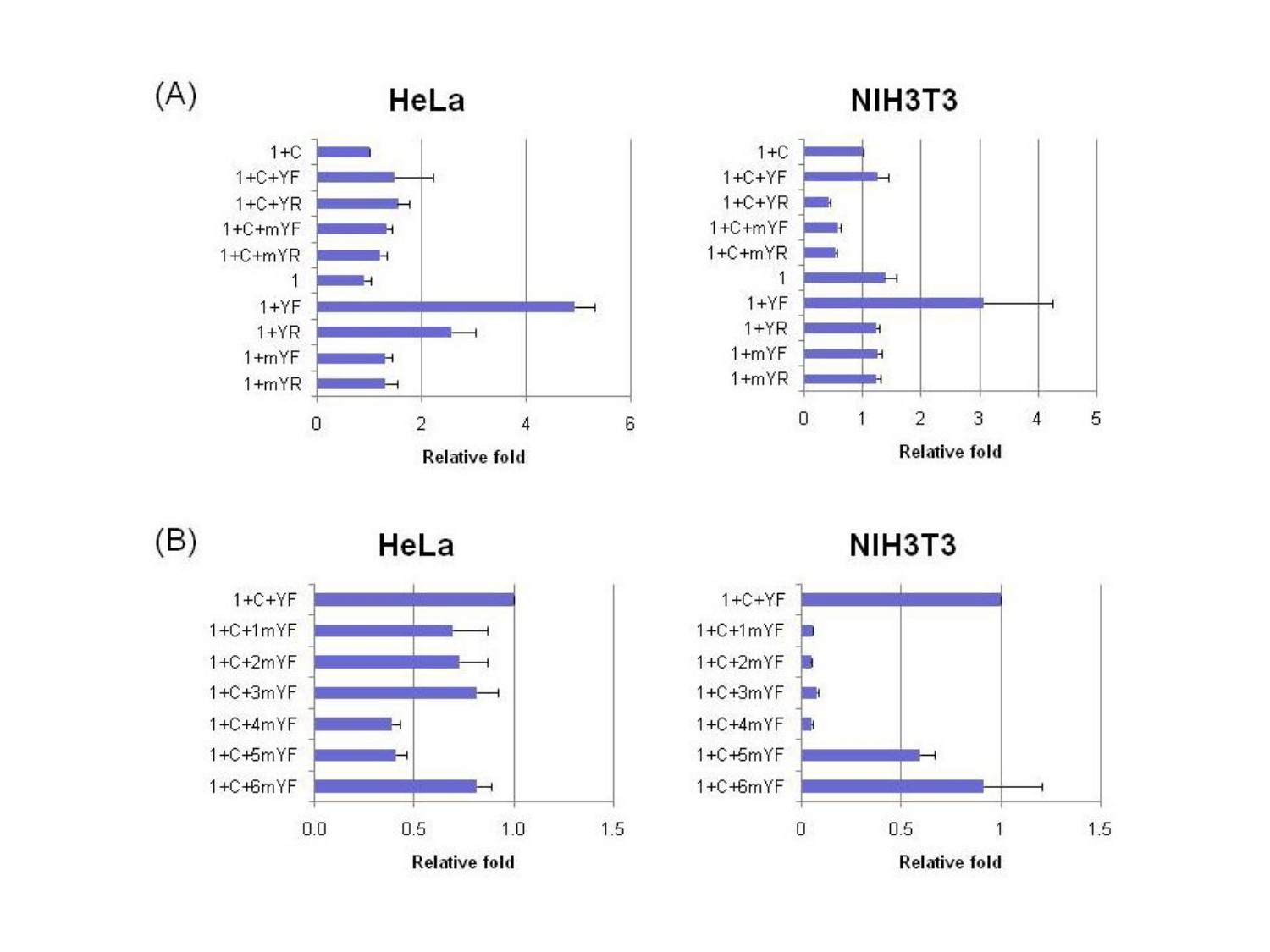

## Slide 2
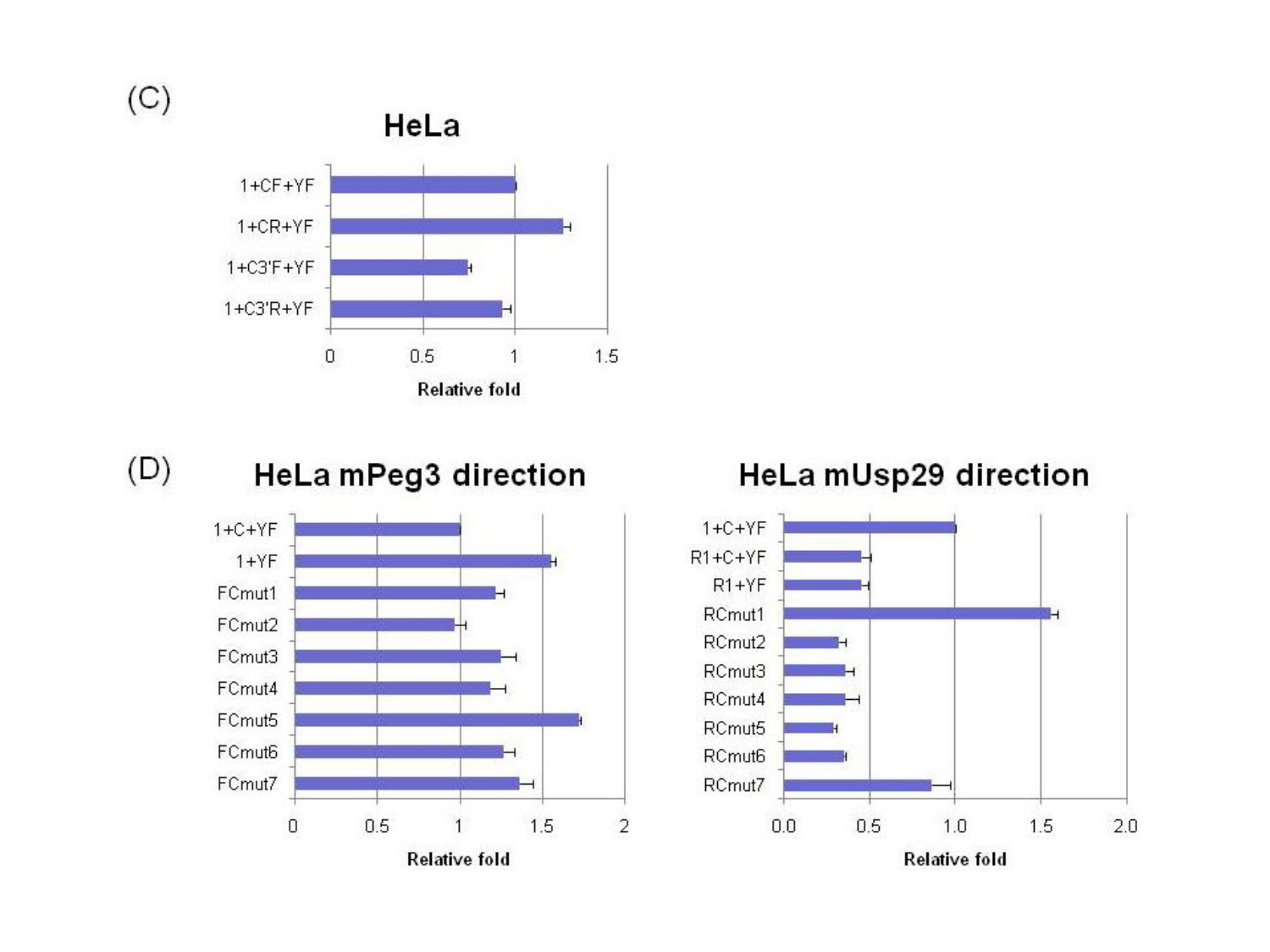

Supplement: Additional file 1 — Transcriptional activity assay. These results derived from other cell lines, HeLa and NIH3T3, using the same construct set presented in Figs. 1A and 3A(A), Fig. 1B(B), Fig. 3B(C), and Fig. 4C(D). [file 1471-2199-9-108-S1.ppt]
